# TRPV4 associates environmental temperature and sex determination in the American alligator

**DOI:** 10.1038/srep18581

**Published:** 2015-12-18

**Authors:** Ryohei Yatsu, Shinichi Miyagawa, Satomi Kohno, Shigeru Saito, Russell H. Lowers, Yukiko Ogino, Naomi Fukuta, Yoshinao Katsu, Yasuhiko Ohta, Makoto Tominaga, Louis J. Guillette Jr, Taisen Iguchi

**Affiliations:** 1 Department of Basic Biology, SOKENDAI (The Graduate University for Advanced Studies), Okazaki Aichi 444-8787 Japan; 2 Okazaki Institute for Integrative Bioscience, National Institute for Basic Biology, National Institutes of Natural Sciences, Okazaki Aichi 444-8787 Japan; 3 Department of Obstetrics and Gynecology, Medical University of South Carolina, and Marine Biomedicine and Environmental Science Center, Hollings Marine Laboratory, Charleston SC 29412 USA; 4 Okazaki Institute for Integrative Bioscience, National Institute for Physiological Sciences, National Institutes of Natural Sciences, Okazaki Aichi 444-8787 Japan; 5 Department of Physiological Sciences, SOKENDAI (The Graduate University for Advanced Studies), Okazaki Aichi 444-8787 Japan; 6 Innovative Health Applications, Kennedy Space Center, Merritt Island FL 32899 USA; 7 Graduate School of Life Science and Department of Biological Sciences, Hokkaido University, Sapporo Hokkaido 062-8520 Japan; 8 Department of Veterinary Medicine, Faculty of Agriculture, Tottori University, Koyama Tottori 680-8553 Japan

## Abstract

Temperature-dependent sex determination (TSD), commonly found among reptiles, is a sex determination mode in which the incubation temperature during a critical temperature sensitive period (TSP) determines sexual fate of the individual rather than the individual’s genotypic background. In the American alligator (*Alligator mississippiensis*), eggs incubated during the TSP at 33 °C (male producing temperature: MPT) yields male offspring, whereas incubation temperatures below 30 °C (female producing temperature: FPT) lead to female offspring. However, many of the details of the underlying molecular mechanism remains elusive, and the molecular link between environmental temperature and sex determination pathway is yet to be elucidated. Here we show the alligator TRPV4 ortholog (AmTRPV4) to be activated at temperatures proximate to the TSD-related temperature in alligators, and using pharmacological exposure, we show that AmTRPV4 channel activity affects gene expression patterns associated with male differentiation. This is the first experimental demonstration of a link between a well-described thermo-sensory mechanism, TRPV4 channel, and its potential role in regulation of TSD in vertebrates, shedding unique new light on the elusive TSD molecular mechanism.

Highly diverse modes of sex determination have been observed among vertebrates. In most cases, sex is determined genetically (genotypic sex determination; GSD), which has been characterized by the presence of a key sex-determining gene. In contrast, in many reptiles including crocodilians, select chelonians and squamates, differential sexual outcomes have been identified under varying incubation temperatures during a critical temperature sensitive period (TSP) in the developing embryo (temperature-dependent sex determination; TSD)^1^,^2^,^3^. As an alternative to the GSD system, in which the heritable traits dictate subsequent gonadal differentiation, all species studied to date that exhibit TSD appear to lack sexually heteromorphic chromosomes^4^,^5^, and sexual development is assumed to be initiated mostly by environmental cues, independent of the individual’s genetic background. However, genetic researches in reptiles are particularly hindered by lack of available genetic manipulation techniques, compared to other vertebrate species, and much of the sex determination mechanism in this pivotal vertebrate clade is yet to be understood.

American alligators, *Alligator mississippiensis,* display TSD, and the developing embryo detects a thermal stimulus that apparently directs the sexual fate of the bipotential gonad; this critical range of incubation temperatures is shared among all other crocodilians studied to date^6^,^7^,^8^. Eggs incubated at a constant temperature of 33 °C yields 100% male offspring, whereas incubation temperatures below 30 °C lead to female biased offspring sex ratio during TSP (Ferguson stages 21 to 24)^9^. Downstream sexual differentiation processes in alligators follows a fairly similar pattern often shared among vertebrates. Male producing temperatures (MPT) facilitates the medullary supporting cells to enlarge and proliferate, which in turn initiates their arrangement into distinct seminiferous cords by hatching (stage 27), and eventual testicular morphogenesis^10^. Dynamic anti-Müllerian hormone (*AMH*) upregulation and ensuing SRY-box 9 (*SOX9*) upregulation are observed during the sex determination phase^11^. At female producing temperatures (FPT), however, primordial germ cell proliferation occurs in the thickened cortex while the medulla undergoes extensive fragmentation^10^,^12^. By developmental stage 27, irreversible female commitment occurs with the eventual onset of estradiol-17β synthesis by aromatase (*CYP19A1*), thus completing ovarian morphogenesis^12^.

However, it remains elusive how incubation temperature during the TSP triggers TSD and the subsequent differentiation cascade. Transformation of the initial environmental temperature signal to a biochemical signaling in TSD is not understood in any species, including species with temperature-dependent sex reversal^13^. Isolated gonads from a TSD reptile, *Trachemys scripta*, have been demonstrated to be directly receptive to thermal stimuli, suggesting that the initial reception of environmental cues can be triggered through an endogenous sensory mechanism^14^. This mechanism hypothesized to be shared among TSD reptiles, including in the alligator as well.

Various mechanisms of thermal detection have been reported in the past, these include protein conformational changes, structural shifts in nucleic acid, and membrane property changes. In eukaryotes especially, several multi-pass transmembrane ion channels have been the focus for thermal sensing^15^,^16^, most prominent of which is the transient receptor potential (TRP) cation channel superfamily. The channels in this superfamily have been the most extensively characterized among vertebrates. The TRP channels in this superfamily mostly function as environmental sensors primarily through Ca^2+^ signaling, uniquely activated by various internal or external cues including osmolarity, pH, pressure, and temperature^17^. At present, 10 thermosensitive TRP channels have been identified in humans and rodents: TRPV1, TRPV2, TRPV3, TRPV4, TRPM2, TRPM3, TRPM4, TRPM5, TRPM8, and TRPA1. Many of the thermosensitive TRP channels possess well-defined ranges of activation as well as wide diversification in physiological and functional properties^18^, and are an ideal candidate as potential thermosensor within TSD mechanism, particularly the TRPV4 since the mammalian TRPV4 channel is known to be activated by moderate heat (27–35 °C)^19^,^20^.

Here, we report the involvement of *A. mississippiensis* TRPV4 (AmTRPV4) ortholog in temperature-dependent sex determination. Electrophysiological analysis reveals AmTRPV4 functions as a molecular thermal sensor at a threshold near the range observed in TSD for this species, and pharmacological manipulation of channel activity affects the sexual differentiation processes in spite of incubation temperature during development. This is the first demonstration of a link between a well-described thermo-sensory mechanism, TRP channel, and regulation of TSD, shedding light on the elusive TSD molecular mechanism.

## Results

### Expression and cloning of AmTRPV4

**Figure 1:**
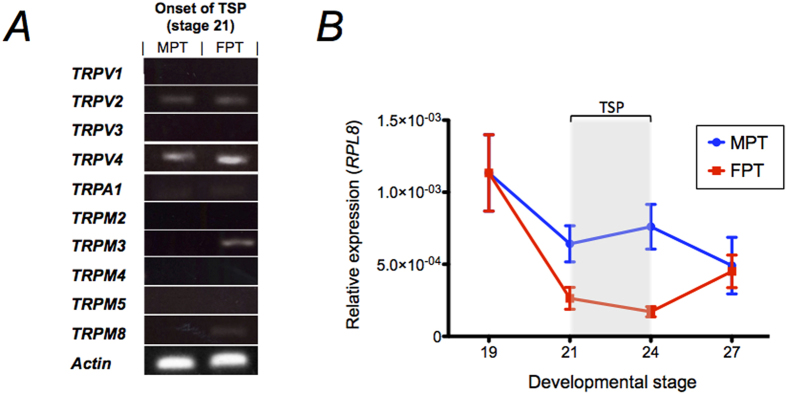
Developmental expression profile of American alligator TRP channels in gonad during sexual development. (***A***) The mRNA levels of various thermosensitive TRP channels were assessed in gonads at the onset of TSP (stage 21) incubated under MPT and FPT conditions. Gene expressions of 5 AmTRP ion channels (AmTRPV2, AmTRPV4, AmTRPA1, AmTRPM3, AmTRPM8) were observed in varying expression levels. (***B***) Quantitative RT-PCR analysis was performed for AmTRPV4 at various key sexual developmental stages including bipotential (stage 19; n = 13), sex determination (stage 21; n = 14, 14), sex differentiation (stage 24; n = 14, 15), and pre-hatching (stage 27; n = 14, 15) stages at both FPT and MPT temperature conditions respectively; ± SEM. Temperature sensitive period is indicated in gray.

To examine the presence of thermosensitive TRP channels in the gonad during the sex determination period in the American alligator, expression for orthologs of the mammalian thermosensitive TRP channels was screened at the onset of TSP and sex determination (Ferguson stage 21) using RT-PCR. Gene expression was limited to 5 TRP ion channels: *TRPV2*, *TRPV4*, *TRPM3*, and faintly from *TRPA1* and *TRPM8* (Fig. 1A). Of the 5 confirmed TRP channels expressed in the gonad, *TRPV4* expression was of particular interest due to ideal predicted activation temperature. Thus, AmTRPV4 was deemed noteworthy for further investigation. Quantitative RT-PCR analysis for *TRPV4* was conducted during various developmental stages; at the bipotential stage (stage 19), onset of TSP and sex determination (stage 21), end of TSP and onset of sexual differentiation (stage 24), and latent stages of sex differentiation (stage 27) at both MPT and FPT. The time series revealed a sexually dimorphic expression patterns in the gonad, with suppression at the FPT (Fig. 1B). *AmTRPV4* expression was also examined in the chorioallantoic membrane, as well as in the epidermal tissues, and its expression was confirmed, although a sexually dimorphic pattern was not observed (Fig. S1A,B).

AmTRPV4 clone (857 aa) was amplified, slightly shorter in comparison to other reported reptilian TRPV4 orthologs, and phylogenetic analysis showed the AmTRPV4 to be more closely related to birds and other reptilian TRPV4 orthologs, when compared to mammalian TRPV4s (Fig. S2). An evolutionary comparison of various TRPV4 orthologs seemingly points toward overall sequence conservation among the higher vertebrates (Fig. S3). Overall similarity in amino acid sequences were observed between alligator TRPV4 and mouse (*Mus musculus*; 86%), human (*Homo sapiens*; 85%), chicken (*Gallus gallus*; 87%), lizard (*Takydromus tachydromoides*; 88%), and snake (*Elaphe quadrivirgata*; 88%)^21^,^22^,^23^. Aside from the proline rich domain (PRD), major domains in the TRPV4 channel structure shared high amino acid sequence identity among higher vertebrate orthologs, and hence, we expected a similar temperature-induced channel activation pattern in AmTRPV4 as observed from mammalian TRPV4 channel activation.

### AmTRPV4 is a potential candidate in TSD initiation

**Figure 2:**
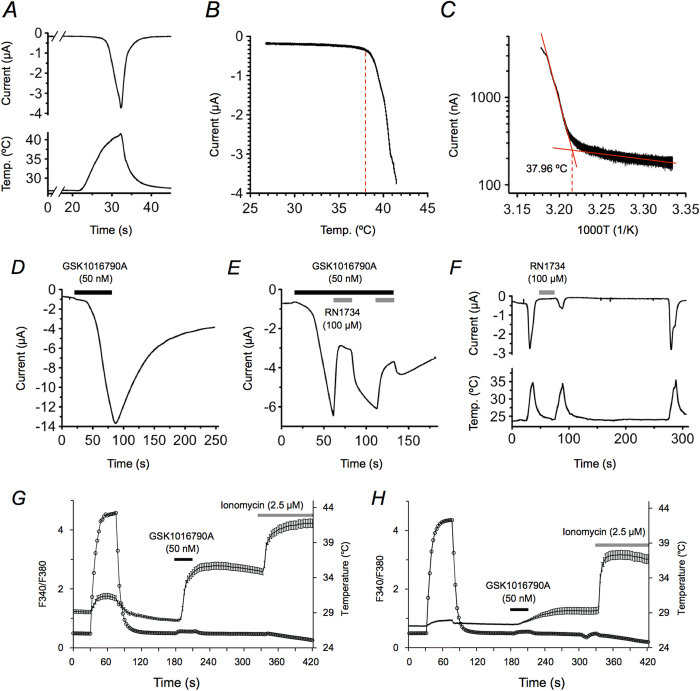
AmTRPV4 is a thermosensitive TRP channel that activates near alligator TSD temperature range. (***A***) A representative trace of the current (upper) activated in response to corresponding changes in bath solution temperature (lower) in the *Xenopus* oocytes expressing AmTRPV4 using a two-electrode voltage-clamp method. (***B***) A representative temperature-response profile for AmTRPV4 activation by heat. (***C***) A representative Arrhenius plot for heat-induced AmTRPV4 activation. The average threshold for activation was 37.30 ± 0.54 °C; n = 17. (***D***) A representative trace of the AmTRPV4 current in the oocyte activated by a TRPV4 agonist (GSK1016790A indicated in a black bar); n = 4. (***E***) A representative trace of AmTRPV4 current in the oocyte activated by administration of a specific TRPV4 agonist (GSK1016790A indicated by a black bar) and subsequently inhibited by TRPV4 specific antagonist (RN1734 indicated by a gray bar); n = 4. (***F***) A representative current trace of AmTRPV4 expressing oocyte activated by heat stimulus and subsequently inhibited by a specific TRPV4 antagonist (RN1734 indicated by a gray bar); n = 4. (***G***) A representative averaged changes of [Ca^2+^]_i_ in AmTRPV4-expressing HEK293 cells (n = 75) under both heat and chemical stimulation. [Ca^2+^]_i_ changes in AmTRPV4-expressing cells (indicated as an average trace ± SE; left y-axis) were observed along with temperatures (indicated by open circle trace; right y-axis). Applications of a TRPV4 agonist (GSK1016790A) and ionomycin are shown with a black and gray bars, respectively. (*H*) Representative [Ca^2+^]_i_ and temperature changes in mock transfected HEK293 cells (n = 36).

At present, very little of the TRPV4 channel thermosensitivity properties have been well characterized in non-mammalian vertebrate species, and hence, characterization of AmTRPV4 was essential before further investigation. Following AmTRPV4 isolation, ion channel functional properties and activation threshold against thermal stimulus were assessed using the *Xenopus laevis* oocyte expression system^24^. Administration of mammalian TRPV4-specific agonist elicited a clear response in cRNA-injected oocytes, and not in negative control (water injected oocyte), indicating successful expression in the oocyte. Furthermore, thermal sensitivity was also confirmed and heat stimulation successfully elicited clear inward current (Fig. 2A,B). *Xenopus* oocytes subjected to water (mock) injection showed no heat-induced current, suggesting specific heat activity by AmTRPV4. An Arrhenius plot analysis indicated an average temperature threshold as 37.30 ± 0.54 °C (n = 17), revealing a warm temperature threshold (Fig. 2C).

Chemical responsiveness to mammalian TRPV4-specific agonists and antagonist administration was examined. AmTRPV4-expressing oocytes displayed current flow with perfusion of GSK1016790A, a potent TRPV4 specific agonist^25^,^26^, at a dose of 50 nM (Fig. 2D). In addition, RN1734, a known TRPV4-specific antagonist^26^, was able to partially and reversibly inhibit AmTRPV4 activated by GSK1016790A (Fig. 2E). Furthermore, RN1734 also was able to inhibit temperature-induced currents in AmTRPV4-expressing oocytes in a reversible manner (Fig. 2F).

AmTRPV4 was next expressed in HEK293 cells and Ca^2+^ imaging experiments were performed to examine whether activation of AmTRPV4 is capable of increasing intracellular Ca^2+^ concentration ([Ca^2+^]_i_). [Ca^2+^]_i_ increased during a heat stimulation above room temperature, as well as after administration of GSK1016790A (Fig. 2G). In contrast, mock-transfected HEK293 cells showed only faint responses to both stimuli (Fig. 2H), suggesting that the Ca^2+^ influx was specifically mediated by the AmTRPV4 channel activation. These results confirmed the sensitivity of AmTRPV4 to warm temperatures, and its responses to chemicals (an agonist and antagonist) were found to be similar to that described for mammalian TRPV4.

### Inhibition of AmTRPV4 during sex determination alters male determination and differentiation-related gene expression

**Figure 3:**
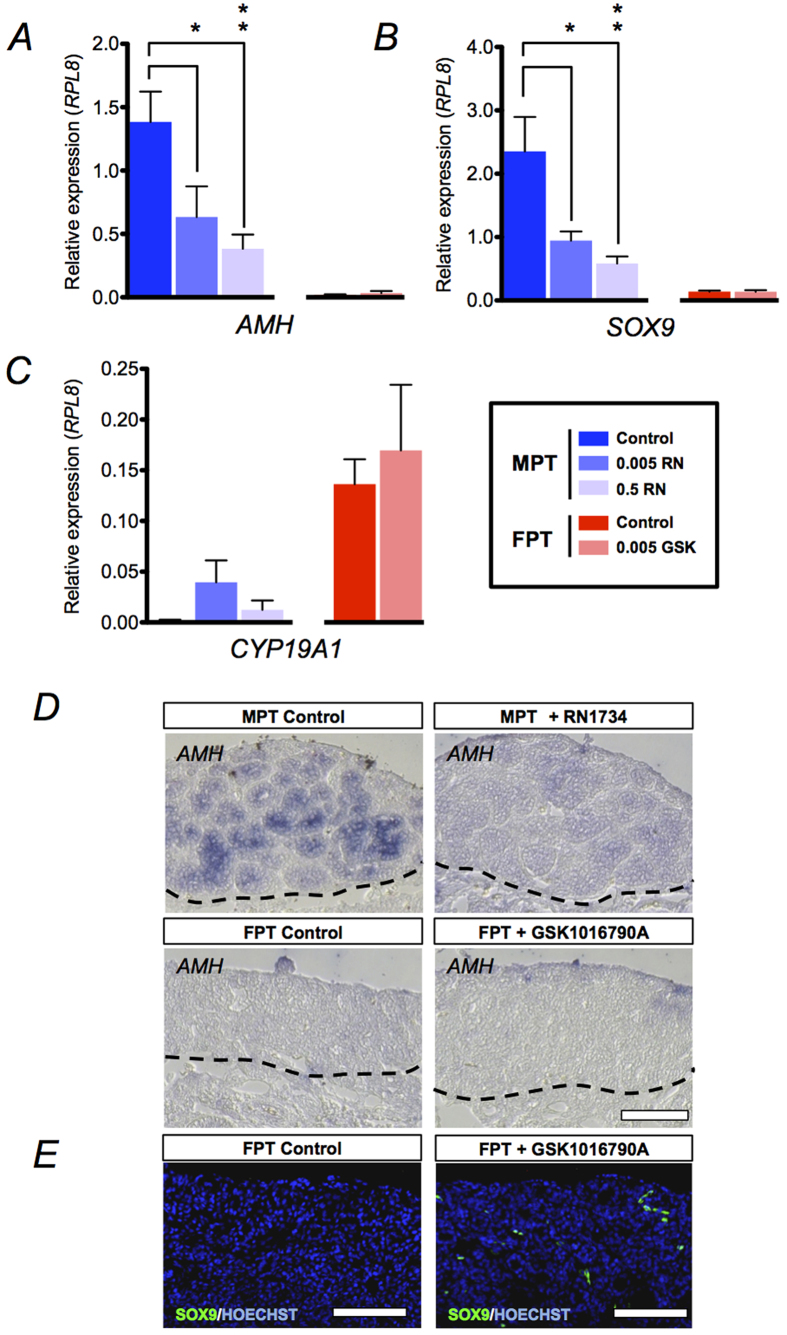
Pharmaceutical activation and inhibition of AmTRPV4 during sex determination alters male differentiation. Stage 19 embryos were administered AmTRPV4 antagonist RN1734 (0.5, 0.005 μg/g egg) or agonist GSK1016790A (0.5, 0.005 μg/g egg) *in ovo* and incubated under MPT and FPT conditions, respectively, until stage 27. (***A*****–*****E***) The mRNA levels of major sex differentiation genes, (***A***) *AMH*, (***B***) *SOX9*, and (***C***) *CYP19A1* in the gonad at stage 27 were examined using quantitative RT-PCR analysis for each treatment: MPT control (n = 12), 0.005 RN (n = 13), 0.5 RN (n = 12), FPT control (n = 13), 0.005 GSK (n = 15). Asterisks indicate statistically significant change in expression; ± SEM; *P ≤ 0.05; **P ≤ 0.01. Markedly lower mRNA expression was observed for *AMH* and *SOX9*, both involved with male differentiation cascade. (***D***) *In situ* hybridization was performed on gonadal cross sections using *AMH* antisense riboprobe. White bar indicates 100 μm. (***E***) Immunohistochemistry for SOX9 and Hoechst was performed on gonad cross sections. White bar indicates 100 μm.

Marked expression of AmTRPV4 in the gonad during TSP, as well as heat-dependent channel activation at a temperature proximate to temperature range involved with alligator TSD provide strong evidence for a possible role of AmTRPV4 in TSD. In order to assess the role of AmTRPV4 during TSD, the channel was evaluated via pharmacological manipulation. Alligator eggs were given a single administration of the chemical agonist GSK1016790A or antagonist RN1734 *in ovo* at stage 19 (bipotential gonad stage), using two different concentrations (0.005 μg/g/egg, and 0.5 μg/g/egg). These doses should be considered as nominal, as we lack information concerning the chemicals’ permeation of the eggshell and half-life *in vivo.* Also, by dosing AmTRPV4 agonist and antagonist to the whole embryo via *in ovo* exposure, we were able to observe potential full body effects following activation or inhibition of AmTRPV4, replicating an elevated or low thermal effect. The eggs were incubated under MPT (33.5 °C) or FPT (30.0 °C) conditions and subsequent effects were examined at stage 27 (stage prior to hatching), focusing on various sex differentiation related genes, and specifically on *AMH*, *SOX9*, and *CYP19A1* gonadal gene expression as sexual markers (Fig. 3A,B,C, Fig. S4A,B).

Quantitative RT-PCR analysis revealed that two genes (*AMH* and *SOX9*) related to testicular differentiation were significantly down-regulated by administration of the AmTRPV4 antagonist RN1734 at MPT conditions in a dose-dependent manner (Fig. 3A,B). Recorded body weights of the embryos were similar among all experimental groups, and the differential expressions were not due to delayed embryonic development^10^ (Fig. S5). Similarly, expression levels assessed by *in situ* hybridization on the differentiated gonads also reflected the results from quantitative RT-PCR, and the lowered gene expression level of *AMH* was confirmed (Fig. 3B). Administration of an AmTRPV4 agonist, GSK1016790A, at FPT did not result in a significant change in gene expression levels based on quantitative RT-PCR, possibly due to sexually dimorphic *AmTRPV4* expression (reduced expression at female generating temperatures) as reported above (Figs. 1B and 3A,C,D). Interestingly, upon closer inspection, the immunohistochemistry revealed an ectopic upregulation of *SOX9* in agonist-treated FPT gonads, indicating that AmTRPV4 activation initiated expression of one of the genes required for male sex differentiation (Fig. 3E). It should be noted, however, that administration of GSK1016790A at the higher dosage induced high mortality; necropsy data indicated premature embryo death that occurred shortly after drug administration. Hence, only low dosage results were available for analysis of the TRPV4 agonist exposure group. In contrast to the genes primarily associated with testicular differentiation, expression levels for *CYP19A1* was unaffected regardless of altered AmTRPV4 channel activity with differing thermal environments (Fig. 3C). As a result, due to a lack of significantly altered *CYP19A1* expression, feminization of RN1734-treated embryos incubated at MPT was very limited: that is, we were not able to induce a feminized gene expression pattern in the gonad following inhibition of AmTRPV4 suggesting that this channel is not effective in the ovarian pathway.

**Figure 4:**
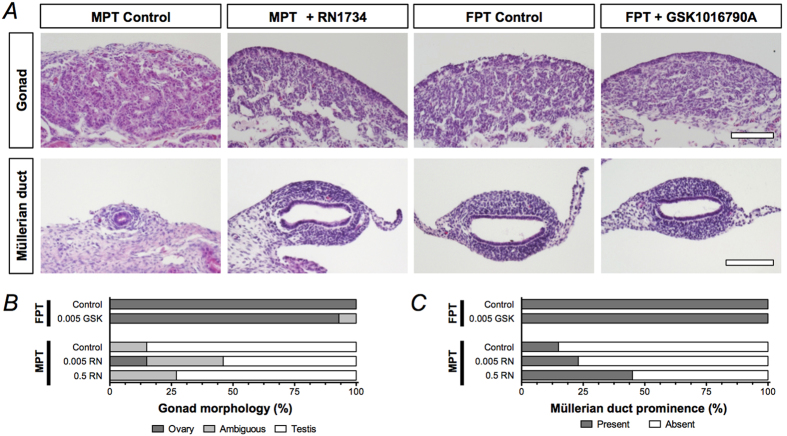
AmTRPV4 inhibition causes rise in Müllerian duct development in MPT. (***A***) Histological analysis of sexual development was performed. Cross-sections of HE stained gonad and Müllerian duct at stage 27 for FPT control (n = 11), 0.005 GSK (0.005 µg/g egg; n = 15), MPT control (n = 13), 0.005 RN (0.005 µg/g egg; n = 11), and 0.5 RN (0.5 µg/g egg; n = 11). Instances of ovarian development were observed in RN1734-treated groups. (***B***) Graph showing number of individuals with ovarian, testicular, or ambiguous morphology in each treatment groups. (***C***) Graph showing number of individuals with prominent Müllerian duct in each treatment groups. White bar indicates 100 μm.

Histological analysis (Fig. 4A,B) revealed that while there were instances of complete feminization following exposure to RN1734, over all both RN1734- and GSK1016790A-treated samples were histologically and morphologically similar to their respective control groups despite significantly lowered *AMH* and *SOX9* gene expression levels in the RN1734-treated groups. However, RN1734-treated groups displayed an increase in prominent Müllerian ducts in a dosage-dependent manner, consistent with lowered *AMH* expression (Fig. 4A,C). In some of these individuals, the ducts showed remarkable signs of regression, namely the reduction in the mesosalpinx^27^. Although the AmTRPV4 targeted treatment did not yield statistically significant phenotypic changes, it did result in a rise of individuals with an abnormal sexual phenotype, with both male-like (testis-like gonad) and female-like (developed Müllerian duct) characteristics.

In summary, manipulation of AmTRPV4 activity impeded induction of the testicular differentiation cascade on a molecular level, but had little effect on the ovarian differentiation cascade, suggesting that TRPV4 does not solely account for thermosensitive trigger mechanism in TSD, but rather, may be part of a larger, more complex mechanism in place.

## Discussion

Here, we demonstrate an active involvement of TRPV4 channel during TSD in *A. mississippiensis*, and its possible role for promoting male development in a temperature-dependent manner. This is the first experimental demonstration of a link between a well-described thermo-sensory mechanism, TRPV4 channel, and regulation of TSD. In many of the environmentally sex determination (ESD) models, it is widely accepted that a presence of an environmental sensor-like element is responsible for the initiation of sex determination cascade. Similarly, several candidate factors have been investigated in TSD species in the past, including epigenetic influences^28^,^29^, heat shock proteins^30^, cold inducible RNA binding protein^3^,^30^, and enzymes related to endocrine signaling such as estrogen^31^,^32^ and/or glucocorticoid^33^,^34^. However, no link between sex determination pathway and temperature sensation could be experimentally demonstrated through these factors and they remained mostly speculations based on correlative data. Our findings serve as a first step toward shedding a new light on the underlying thermosensitive mechanism.

Our results indicate that while AmTRPV4 channel activity may significantly influence male gonadal sex determination pathway at a molecular level, it alone does not account for the initiation of gonadal sex determination mechanism, as evidenced by lack of significant gonadal sex reversal due to unaltered *CYP19A1* expression, and seemingly independent female gonad sex determination pathway. Rather, TPRV4 is expected to be a component of the much larger framework governing the initiation of TSD. Ambient temperature can broadly influence multiple targets at once and most likely several elements are involved in the TSD mechanism one way or the other, including the potential factors investigated previously. Furthermore, co-regulation of Ca^2+^ signaling by multiple TRP channels has been well described before^35^, and other TRP channels observed could also be involved. Although current study employs pharmacological manipulation as a first step to study AmTRPV4’s role in TSD, more sophisticated methodology is desired in order to evaluate the relational extent between TRPV4 and TSD in the near future.

Our results also indicate that alligator TRPV4 channel activates at 37.3 °C, higher than we had originally anticipated, that is, artificial incubation at a constant temperature of 35 °C or higher have been associated with embryonic death in the past studies^6^,^8^. In the wild, however, we regularly recorded nest temperatures in that range from nests that produce viable hatchlings (Fig. S6). We observed that nest temperature fluctuates daily and during incubation in natural alligator nest, and many nests exhibited elevated temperatures, suggesting thermal patterns during incubation may be critical as well.

Although mammalian TRPV4 activates at 27–35 °C, AmTRPV4 showed activation at a relatively higher temperature, near 37 °C, based on our results. Evolutionary shift in temperature sensitivity has been observed among thermosensitive TRP channel orthologs from various vertebrate species and would most likely account for the difference observed in AmTRPV4. How much of our results from the *Xenopus* oocyte *in vitro* experiment translates to the actual embryonic environment during TSP are up to debate. Although MPT (33.5 °C) is relatively lower than supposed activation temperature, *in ovo* administration experiment showed that inhibiting AmTRPV4 activity was still able to induce significant differential gene expression. Activation temperatures of TRP channels have been reported to shift depending on the cellular environment^20^. In the case of TRPV4, membrane properties greatly influence thermal sensitivity^20^,^36^, and a delicate difference in the membrane environment may account for the 4 °C margin of difference in this case.

A relatively small degree of sexual dimorphism in *AmTRPV4* gene expression helps explain why the molecular mechanisms behind TSD are so difficult to identify; unlike GSD, in which dynamic sexually dimorphic gene expression (e.g., mammalian *Sry*, galline *DMRT1*) determines sexual fate, the TSD may not necessary be initiated by dimorphic gene expression alone, as implied from our results with TRPV4 channel activity in alligators. Overdependence on comparative analysis between TSD and GSD models may restrict further discoveries; an independent approach will be required for constructing TSD models in the future. It has been suggested that ‘cumulative discrepancy’ underlies TSD, as implied in many of the TSD reptiles^37^, and activation of AmTRPV4 during the lengthy TSP may contribute to such cumulative mechanism.

Multiple functions have been attributed to the mammalian TRPV4, a polymodal Ca^2+^-permeable channel, including cell-death induction, alteration of gene expression, channel trafficking, and protein interactions by careful maintenance of Ca^2+^ levels^38^. This highly flexible and adaptable nature is suitable for regulating cell fate; TRPV4 plays a crucial role in various cellular differentiations, such as chondrocyte^39^, myofibroblast^40^, osteoclast^41^, and keratinocyte^42^. TRPV4-assisted steady influx of Ca^2+^ allows for Ca^2+^ /calmodulin (CaM) complex-mediated molecular cascades to take place, such as *Sox9* upregulation in chondrocyte differentiation^38^,^39^. Consistent with previous reports, AmTRPV4-mediated Ca^2+^ influx through channel activation was confirmed, and the positive relationship observed between AmTRPV4 channel activity and *SOX9* expression from our results resembled the regulatory relationship reported during chondrocyte differentiation. TRPV4′s role in gonad morphogenesis is less studied, though Ca^2+^ influx is an essential component in mammalian male sex determination^43^,^44^. Indeed, CaM is crucial for the nuclear import of SOX9 and its subsequent transcriptional activity in mammals, and the loss of a SOX9-CaM interaction is associated with autosomal sex reversal (SRA) disease in humans^44^. Additionally, innate male biased sexual dimorphism in TRPV4 channel activity has been reported in several studies involving *Trpv4*-deficient mouse^45^,^46^. Interestingly, van der Eerden *et al.* (2013)^46^ speculated TRPV4 as a male-specific regulator of osteal cell differentiation.

Crocodilians represent an interesting presence among the reptiles. *Alligator mississippiensis* is believed to display a type II TSD pattern^6^,^7^, in which the embryo feminization is attained at both low and extreme high temperatures, although 100% male productions at incubation temperatures as high as 36 °C have also been previously reported^8^. Based on our results, this may even imply a presence of a secondary thermosensitive mechanism, though molecular data concerning high temperature female are scarce, and require more investigation before making further insights. While that, in and of itself, already indicates a complex TSD-triggering mechanism among crocodilians, their sex determination threshold temperature is also substantially higher than majority of TSD reptiles^6^, and the sexual developmental pattern are distinct from other reptile species (e.g., *AMH* expression precedes *SOX9* during testis differentiation)^47^. Variation in the TSD pattern, as well as inconsistency in the gene expression patterns early on in the sex determination even among other TSD reptiles^48^,^49^,^50^, questions the extent of homogeneity and diversity within TSD mechanisms and potential role of TRPV4 in TSD. Non-mammalian vertebrate TRP channels are only starting to be analyzed, including those in reptiles^51^,^52^, and further insight is expected in the near future.

## Materials and Methods

### Animals, tissue collection and chemicals

Alligator eggs were collected at Lake Woodruff National Wildlife Refuge, Volusia County, FL, USA were approved by and under permits from the Florida Fish and Wildlife Conservation Commission and the U.S. Fish and Wildlife Service (Permit #: SPGS-10-44). Alligator eggs were collected in June of 2011 to 2013. All works involving alligators were approved by and was performed under the guidelines specified by the Institutional Animal Care and Use Committee at Medical University of South Carolina (Permit #: AR3036). Once the eggs were collected, they were transported to Hollings Marine Laboratory (Medical University of South Carolina; Charleston, SC, USA) where they were incubated in damp sphagnum moss.

Embryonic developmental stage of each clutch was determined using criteria described by Ferguson (1985)^9^. Until embryonic stage 19, the eggs were incubated under FPT (30.0 °C), at which point they each underwent random treatments and were separated into two groups, incubated under MPT (33.5 °C) or FPT (30.0 °C). GSK1016790A, (Sigma-Aldrich, St. Louis, MO, USA) and RN1734 (Tocris Bioscience, Bristol, UK) were dissolved in ethanol for embryonic exposure experiment. 0.005, 0.5 μg/g egg of agonist (GSK1016790A) and antagonist (RN1734) were administered once at stage 19 *in ovo*. Ethanol was administrated as vehicle in control group. The alligator embryonic GAM (gonad adrenal mesonephros complex), chorioallantoic membrane, and epidermal tissues were sampled at stages 19, 21, 24 and 27, and subsequently preserved in either RNAlater (Life Technologies, Carlsbad, CA, USA) or 4% paraformaldehyde until further analysis.

### Molecular cloning of AmTRPV4 and sequence analysis

Total RNA was extracted from female neonatal gonad, using RNeasy kit (Qiagen, Valencia, CA, USA). Full coding region of the AmTRPV4 was determined by standard procedure using SmartRACE kit (Takara, Ohtsu, Japan), and finally full-length AmTRPV4 was cloned with KOD + polymerase (Toyobo, Osaka, Japan). The amplified full-length AmTRPV4 cDNA product was then subcloned into pOX + vector^24^ for electrophysiological analysis. Primer information is reported in Table S1.

Multiple sequence alignment for vertebrate TRPV4 homologues was performed using the CLUSTAL W^53^. Phylogenetic relationships of TRPV4 were then examined using TRPV4 amino acid sequences derived from GenBank/EMBL database. The TRPV4 genes and species used are summarized in Table S2. Based on TRPV4 conserved sites, which include the ankyrin repeat domains and transmembrane domains (652 residues in AmTRPV4), with all the alignment gap sites were eliminated, minimum-evolution methods^54^ was applied to construct an evolutionary tree, using MEGA 5 software^55^. The statistical confidence was then computed by bootstrap method with 1000 replications.

### Oocyte electrophysiology

The alligator TRPV4 channel was expressed in the oocytes of the African clawed frog, *Xenopus laevis*, and ionic currents were recorded using the two-electrode voltage-clamp method as described previously^24^. cRNA of the full length AmTRPV4 channel clone inserted between *X. laevis* β-globin 5′ and 3′ UTR in the pOX + vector was synthesized using mMessage mMachine kit (Life Technologies) according to the manufacturer’s protocol, and was injected into *Xenopus* oocytes (4, 10 ng/μl) and ionic currents were recorded 2–5 days post-injection using a heat perfusion system. ND96 solution, which consists of 96 mM NaCl, 2 mM KCl, 1.8 mM CaCl_2_, 1 mM MgCl_2_, and 5 mM 2-[4-(2-Hydroxyethyl)-1-piperazinyl] ethanesulfonic acid (HEPES), was used as bath solution after adjusted to pH 7.4. The oocytes were voltage-clamped at -60 mV. All chemicals used for assay were diluted into ND96 bath solution, and applied to the AmTRPV4 expressing oocytes through perfusion. Likewise, heated thermal stimulations were applied to the oocytes by heated ND96 bath solution perfusion. Temperature threshold for activation was determined by Arrhenius plot, using Origin software (OriginLab, Northhampton, MA, USA). All procedures involving the care and use of the frogs were approved by Institutional Animal Care and Use Committee of National Institutes of Natural Sciences, Japan.

### Ca^2+^-imaging experiments

Alligator TRPV4 was expressed in HEK293 cells, and Ca^2+^ -imaging experiments were performed using methods previously described^24^. HEK293 was cotransfected with AmTRPV4 recombinant pcDNA3.1 + vector and DsRed containing pCMV vector using Effectene Transfection Reagent (QIAGEN) following manufacturer’s protocol. The transfected cells were incubated at 33 °C and then used for Ca^2+^-imaging experiments after incubation lasting ~24 h. Fura-2 was loaded into cells by incubating at 33 °C for 1 h with fura-2 acetoxymethyl ester. During recording, the fura-2-loaded cells were placed in a recording chamber filled with bath solution (140 mM NaCl, 5 mM KCl, 10 mM HEPES, 2 mM MgCl_2_, 2 mM CaCl_2_, 10 mM glucose, pH 7.4). Heated bath solution was perfused for thermal stimulation. For chemical stimulation, GSK1016790A (50 nM) or ionomycin (2.5 μM) were dissolved in bath solution and perfused. [Ca^2+^]_i_ in transfected cells were measured under light at 340 nm and 380 nm, while fluorescent signals at 500 nm were recorded and their ratios (F340/F380) were calculated. The cells expressing DsRed were used for analysis as AmTRPV4 expressing cells. For negative control experiments, HEK293 cells were cotransfected with pcDNA3.1 and DsRed containing pCMV vectors.

### Nest temperature data

One hundred and eighty TidbiT v2 programmable temperature data loggers (Onset Computer, Bourne, MA, USA) were deployed in 48 alligator nests during the 2010–2014 nesting seasons to determine actual nest temperatures within wild American alligator nests. Further information is available on SI Text.

### Expression profiling and statistical methods

Gonadal tissues were carefully dissected from the GAM samples stored in RNAlater. Total RNAs were then extracted from gonadal, chorioallantoic membrane, and epidermal tissues, using SV Total RNA Isolation System (Promega, Madison, WI, USA). Template cDNA was synthesized from purified total RNA with iScript cDNA synthesis kit (Bio-Rad, Hercules, CA, USA). Primers used for TRP expression profiling was validated by confirming TRP expression in various tissues with 35 PCR reaction cycles. Real-time quantitative PCR reactions were performed using ABI Prism 7000 (Life Technologies) using the SYBR-Green PCR core reagents kit (Life Technologies), in the presence of appropriate primers, with *RPL8* as a housekeeping gene. Primers used were constructed based on previously reported sequences^28^,^56^. Primer information is reported in Table S1. Each gene was assayed in triplicate samples with relative standard curve method under the following conditions: 2 min at 50 °C and 10 min at 95 °C, followed by 40 two-temperature cycles (15 sec at 95 °C and 1 min at 60 °C). Data acquisition and analyses were performed by ABI Prism 7000 SDS software ver 1.1 (Life Technologies). The average individual gene expressions were normalized to each average *RPL8* mRNA expression level. The quantitative RT-PCR results are presented as mean ± SEM. Two-way ANOVA was performed for comparisons among temperature responses at different developmental stages in TRPV4 channel expression profiling. Multigroup comparisons between MPT experimental groups were performed using one-way ANOVA, followed by stepwise Tukey-Kramer post hoc adjustments. Comparison between FPT experimental groups was performed using Student’s *t*-test. Statistical analyses were performed using GraphPad Prism (Version 5.0b; GraphPad Software, Inc., San Diego, CA, USA) software. *P*-value lower than 0.05 was considered to be statistically significant.

### Histological analysis

After fixation of GAM tissues in 4% paraformaldehyde, they were dehydrated and embedded in paraffin. GAMs were cross-sectioned at 6 μm, re-hydrated using reverse ethanol gradient, and stained with hematoxylin and eosin for standard HE staining. Morphological changes were assessed using Pearson’s chi-square test of independence. For *in situ* hybridization analysis, Alligator *AMH* riboprobe, using design by Western *et al.* (1999)^47^, was hybridized *in situ* to sections following standard protocols using species-specific digoxigenin-labeled antisense riboprobes at 65 °C. Primer information is reported in Table S1. For immunohistochemistry, sections of deparaffinized GAMs were washed twice in PBS, microwaved in citrate buffer for 10 min. Samples were then incubated with blocking solution consisting of 0.5% Boehringer Blocking reagent (Roche Mannheim, Germany), 10% heat-inactivated fetal bovine serum (FBS), 3% bovine serum albumin (BSA), and 0.2% Triton-X-100 in PBS for 30 min. Sections were incubated with anti-SOX9 antibody (Chemicon AB5535; Temecula, CA, USA) which was reportedly compatible with turtle SOX9^57^ as primary antibody in fresh blocking solution overnight at 4 °C at 1:1000. The samples were then washed three times in fresh blocking solution (as described above, with the exception of using 1% FBS instead) for 30 min, and then blocked for an additional hour with anti-rabbit secondary antibody label Alexa Fluor 488 (Life Technologies) at 1:200 at room temperature. The DNA was stained with Hoechst at 1:1000.

## Additional Information

**How to cite this article**: Yatsu, R. *et al.* TRPV4 associates environmental temperature and sex determination in the American alligator. *Sci. Rep.* **5**, 18581; doi: 10.1038/srep18581 (2015).

## Supplementary Material

Supplementary Information
