# Supplementary material for: TRPV4 associates environmental temperature and sex determination in the American alligator
Source: Sci Rep. 2015 Dec 18;5:18581. doi: 10.1038/srep18581 (PMC4683465; doi:10.1038/srep18581)
Supplement: Supplementary Information [file srep18581-s1.pdf]

TRPV4 associates environmental temperature and sex determination in the American alligator

Ryohei Yatsu[a,b,1], Shinichi Miyagawa[a,b,1] Satomi Kohno[c], Shigeru Saito[d,e], Russell H. Lowers[f], Yukiko Ogino[a,b], Naomi Fukuta[d], Yoshinao Katsu[g], Yasuhiko Ohta[h], Makoto Tominaga[d,e], Louis J. Guillette Jr.[c,]*, and Taisen Iguchi[a,b,]*

Supporting Information

Supporting Figures

Fig. S1.

TRPV4 channels expression in chorioallantoic membrane and epidermal tissues during sexual development. (*A*) Quantitative RT-PCR analysis was performed for AmTRPV4 at various key sexual developmental stages including bipotential (stage 19), sex determination (stage 21), sex differentiation (stage 24), and pre-hatching (stage 27) stages at both temperature conditions, in chorioalantoic membrane tissue and abdominal epidermal tissues; ± SEM. Temperature sensitive period is indicated in gray.

Fig. S2.

Phylogenetic tree of vertebrate TRPV4. Phylogenetic relationship among TRPV4 amino acid sequences for selected higher vertebrate species is shown. Phylogenetic tree was constructed based on conservative domains including ankyrin repeat and transmembrane domain, using minimum evolution method with Jones-Taylor-Thornton model. Bootstrap value is indicated beside the respective branch (Bootstrap values

below 70 percent not shown). Database accession numbers of genes used is tabulated in Supplementary Table 2. Zebrafish TRPV4 was used as outgroup, and Western clawed frog TRPV4b, c, d, e, f is omitted for simplicity.

Fig. S3.

Vertebrate TRPV4 amino acid alignment and identity. (*A*) Alignment of TRPV4 amino acid sequences from various vertebrates TRPV4; overall similarity in amino acid sequences were observed between alligator TRPV4 and mouse (*Mus musculus*; 86%), human (*Homo sapiens*; 85%), chicken (*Gallus gallus*; 87%), lizard (*Takydromous tachydromoides*; 88%), and snake (*Elaphe quadrivirgata*; 88%) TRPV4. Major channel structures are indicated with a bar, including proline-rich domain (PRD), ankyrin repeat domain (ARD), transmembrane region (TM), pore loop (PL), TRP domain (TRP), and calmodulin binding domain (CaMBD). Numbers indicate amino acid residue position. The alignment was created using ClustalW. (*B*) Schematic for the major functional structure, and amino acid identity (%) for each of the selected vertebrate TRPV4, in comparison to AmTRPV4.

Fig. S4.

Expression levels of major sexually differentiation genes, DMRT1 and FOXL2. The mRNA levels of major genes related to sexual development, (*A*) Doublesex and mab-3 related transcription factor 1 (*DMRT1*), and (*B*) Forkhead box protein L2 (*FOXL2*), were also examined using quantitative RT-PCR analysis. Trend similar to dosage-dependent down regulation was observed for *DMRT1,* gene involved with male differentiation.

Fig. S5.

Comparison of embryo body weights. Embryonic body weight at the time of sampling (Ferguson stage 27) was recorded for all experimental groups. Treatment of RN1734, TRPV4 antagonist, or GSK1016790A, TRPV4 agonist, on the embryo at MPT and FPT, respectively, did not alter the final body weight, and the no delay in development rate was observed.

Fig S6.

Representative alligator nest temperature data from the wild. The thermisters were placed within the nest touching eggs at the bottom, middle and top of the nest throughout the entire incubation process. Many nests exhibited elevated temperatures throughout the course of incubation, and a relatively hot nest temperature of 35°C or higher was regularly recorded, with viable hatchlings.

Supporting Methods

Nest Temperature data

One hundred and eighty TidbiT v2 programmable temperature data loggers (Onset Computer, Bourne, MA, USA) were deployed in 48 alligator nests during the 2010-2014 nesting seasons to determine actual nest temperatures within wild American alligator nests. The loggers were placed within the nest touching eggs at the bottom, middle and top of the nest throughout the incubation process. A fourth temperature logger was also placed on the outside of the nest hanging in the air above to collect ambient air temperature. The thermistors were programmed to record the temperature

every five min and were picked up either when the nest hatched, or after 75 days of deployment time (whichever came first). Once retrieved, data from the thermistors were downloaded to examine temperature profiles throughout the incubation period as well as during the period of sexual determination.

For thermister deployment, a vertical 25 cm channel next to the nest chamber was dug out on one side of the eggs within the nest. A rebar or wooden stake with all of the thermistors tied to it was inserted into the thermistor chamber farthest away from the eggs as possible to keep the thermistors at the nest site during the removal of the neonates. Starting from the bottom of the eggs in the nest a thermister would be placed touching the bottom egg, with the serial number and depth of placement noted on the data sheet. The nesting material would then be placed on that thermister until the middle of the egg chamber where the second thermister would be placed touching an egg. This process was replicated with the third and/or top thermister. The top of the nest was covered with the natural nesting material and left alone until thermistors were recovered. To verify temperature accuracy, the loggers were placed into an incubator with set temperature shifts to verify the accuracy of all loggers. The last calibration had variability within all of the sensors of 0.16 of a degree of true calibration. Out of the 48 nests in which thermistors were deployed, 32 nests hatched, 10 nests were dead, and 6 were depredated (3 hogs, 1 raccoon and 2 fire ants).

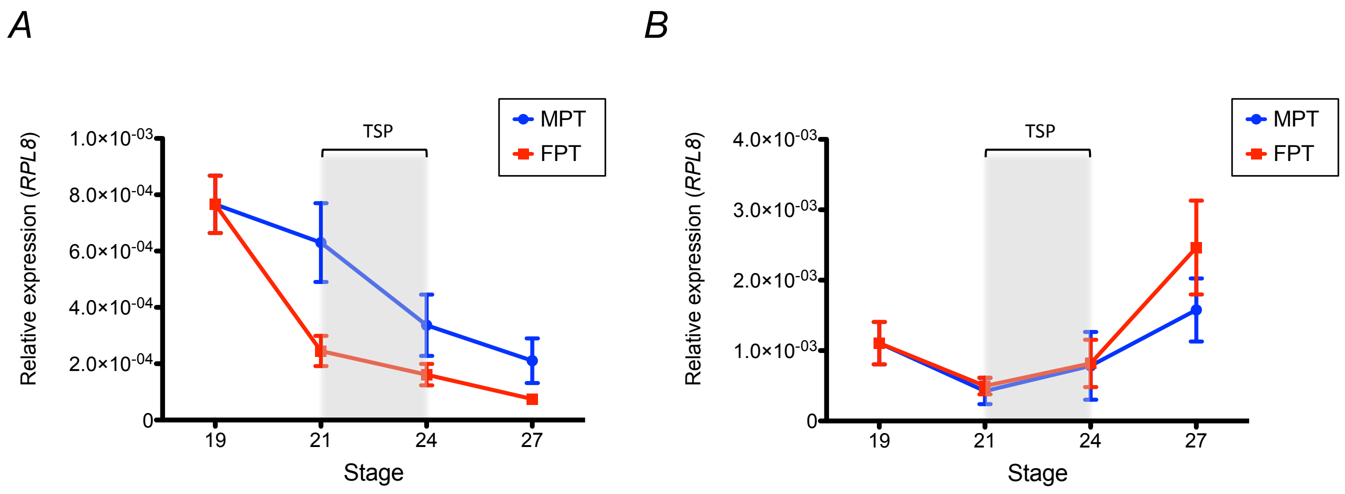
A
B
MPT
FPT
TSP
Relative expression (RPL8)
1.0×10-03
8.0×10-04
6.0×10-04
4.0×10-04
2.0×10-04
0
19
21
24
27
Stage
4.0×10-03
3.0×10-03
2.0×10-03
1.0×10-03

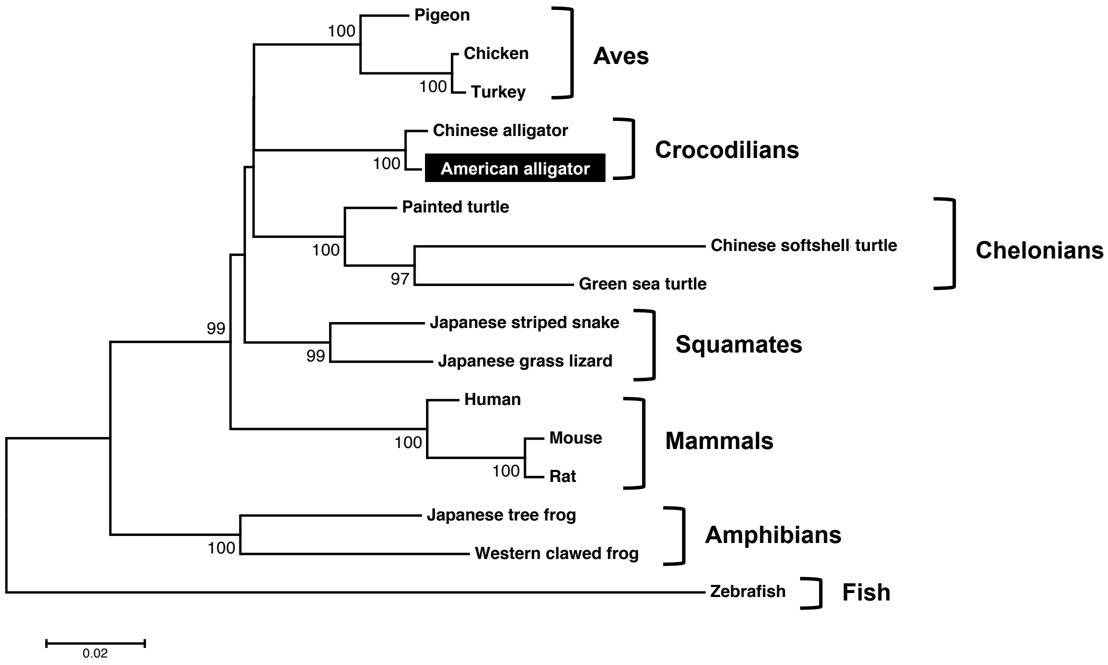
Pigeon
Chicken
Turkey
Aves
Chinese alligator
American alligator
Crocodilians
Painted turtle
Chinese softshell turtle
Green sea turtle
Chelonians
Japanese striped snake
Japanese grass lizard
Squamates
Human
Mouse
Rat
Mammals
Japanese tree frog
Western clawed frog
Amphibians
Zebrafish
Fish
100
100
100
100
97
99
99
100
100
100
0.02

A

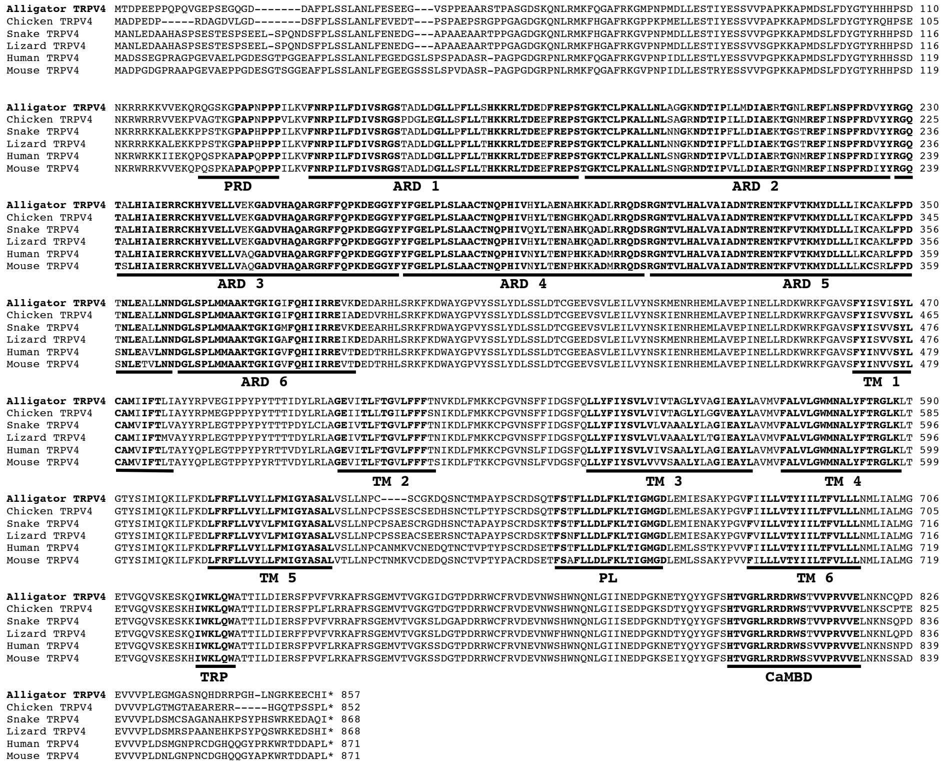

B

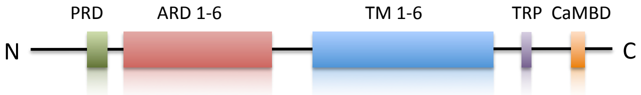

	PRD	ARD 1-6	TM 1-6	TRP	CaMBD
Alligator	100	100	100	100	100
Chicken	91	92	91	100	100
Snake	89	94	89	100	100
Lizard	89	94	88	100	100
Mouse	67	90	85	100	95
Human	67	91	88	100	95

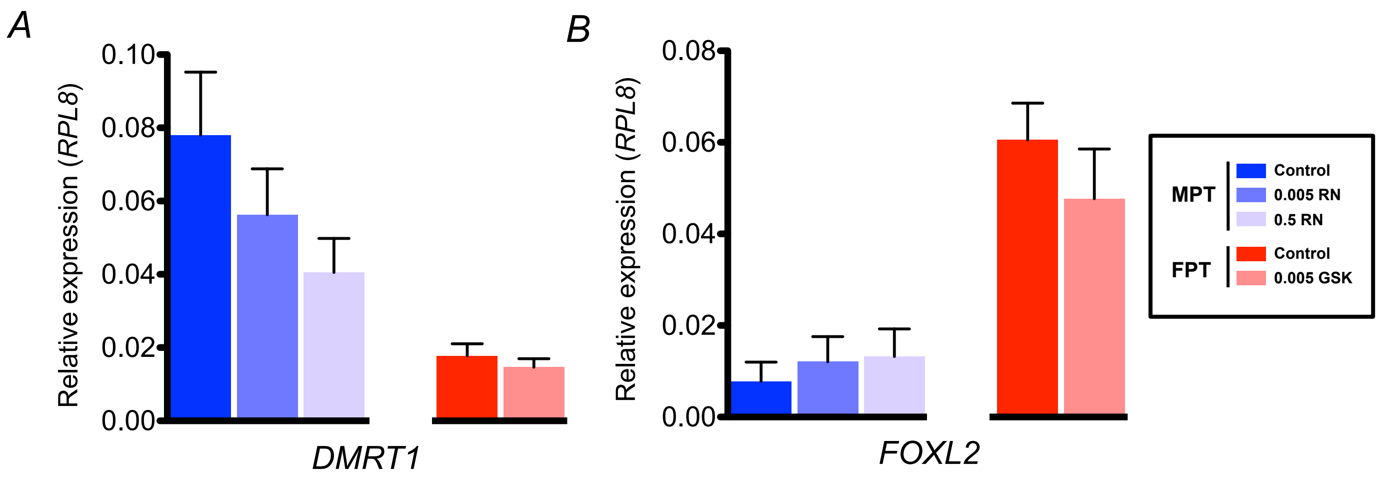
A
Relative expression (RPL8)
0.10
0.08
0.06
0.04
0.02
0.00
DMRT1
B
Relative expression (RPL8)
0.08
0.06
0.04
0.02
0.00
FOXL2
MPT
Control
0.005 RN
0.5 RN
FPT
Control
0.005 GSK

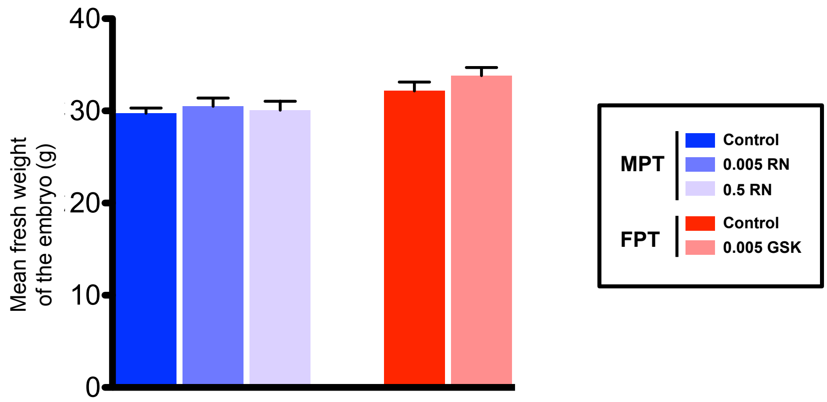

40
30
20
10
0
Mean fresh weight
of the embryo (g)
MPT
Control
0.005 RN
0.5 RN
FPT
Control
0.005 GSK

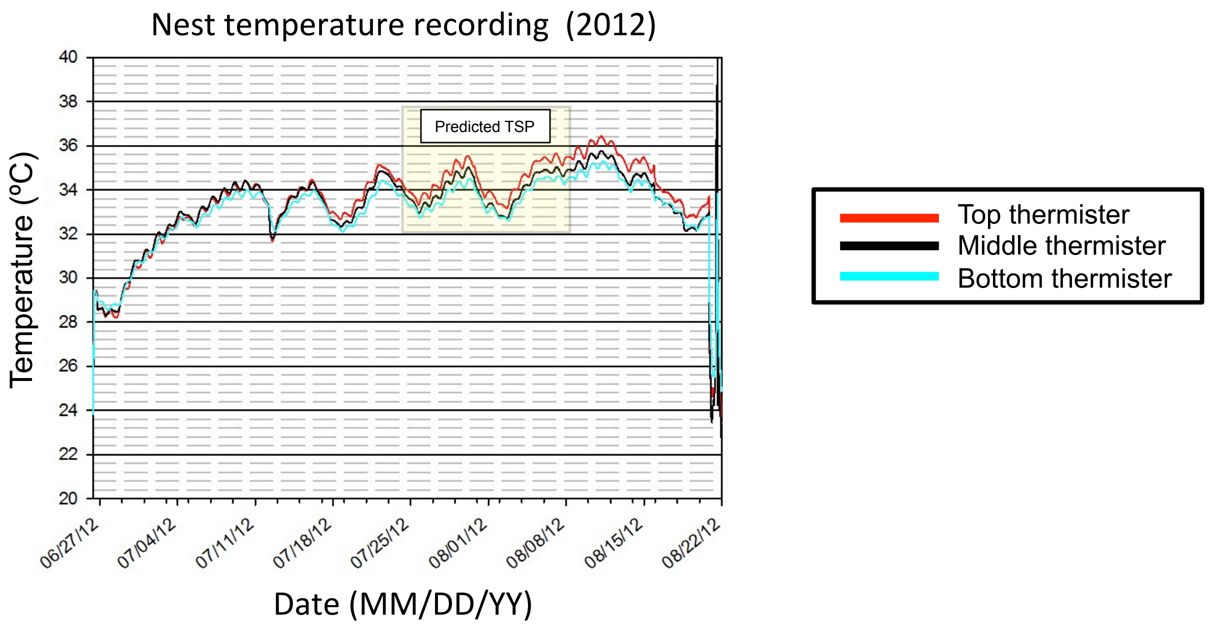

Nest temperature recording (2012)
Temperature (ºC)
40
38
36
34
32
30
28
26
24
22
20
Predicted TSP
06/27/12
07/04/12
07/11/12
07/18/12
07/25/12
08/01/12
08/08/12
08/15/12
08/22/12
Date (MM/DD/YY)
Top thermister
Middle thermister
Bottom thermister

Table S1: Primer information

Name	Sequence	
**TRPV4 cloning and isolation**		
aTRPV4-A:	5’-TTYTTYCARCCNAARGAYGARGGN-3’	
aTRPV4-B:	5’-GGNGGNTAYTTYTAYTTYGGNGAR-3’	
aTRPV4-C:	5’-ACYTGNCCNACNGTYTCNCCCAT-3’	
aTRPV4-D:	5’-GCCCAYTGNARYTTCCADATRTGYTT-3’	
5’RACE-1	5’-TCCCGGGTGTTGTCAGCGATGGCGACC-3’	
5’RACE-2	5’-AGCACGGTGTTGCCGCGGGAGTCCTGG-3’	
3’RACE-1	5’-GAGACGGTGGGGCAGGTGTCCAAGGAG-3’	
3’RACE-2	5’-ATCTGGAAGCTGCAGTGGGCCACCACC-3’	
Cloning	5’-TGTGTGCTGACCCATAACGCGTGCCAG-3’	5’-CCCGGGAGAGGAGAAACATGGTGCAAG-3’
**TRP subtype gene expression RT-PCR**		
TRPV1	5’-GAATCTGAAGTCACCGACGAGCA-3’	5’-AAAGATCCTCCTGCGATCATAGTACTTG-3’
TRPV2	5’-CTCAGTCTTTGCCTACCATTGTCCC-3’	5’-AGACCATCAACGGGGTGTAGTATTC-3’
TRPV3	5’-GCTTATGGGCAAGAAACCAAATCCA-3’	5’-CCCTCAGATACTGCCCTGAACAAATA-3’
TRPV4	5’-GAGTCCACCATCTACGAGTCCTCC-3’	5’-CAGGTTCAGCAGTGCCTTGGG-3’
TRPM2	5’-CTTTTCCGGGACACCTATGAGTTCTT-3’	5’-TCACGAACTCAGGCTTGTTGGAAAT-3’
TRPM3	5’-GTCATTCGGCATGTTGGAGATGC-3’	5’-GTGCTTTTCCAGTTGTCTACGGAGT-3’
TRPM4	5’-TGCGACTCATCCACATCTTCGC-3’	5’-CGAGGAGGAAGACAACAAGGAGAAC-3’
TRPM5	5’-ATGATGCCTGCAAAGGTTTCTACCA-3’	5’-CGGTCTTCGCTGTTGTGATAACATTC-3’
TRPM8	5’-GAAACACACAAAGGAACTCCCTACTAATG-3’	5’-ACATTCTCCTCGGAAGTCCTACTGA-3’
TRPA1	5’-ACAGAAAACGTATAGCAGTCCACTCC-3’	5’-CAATATGGCAGCTTCTTCTCTAAGTTTGT-3’
**Quantitative RT-PCR primer**		
RPL8	5’-GGTGTGGCTATGAATCCTGT-3’	5’-ACGACGAGCAGCAATAAGAC-3’
TRPV4	5’-TCACCTTCGTGCTGCTGCTT-3’	5’-AGATCTGCTTGCTCTCCTTG-3’
SOX9	5’-AGTACCCCCATCTGCACAAC-3’	5’-CCCGTTCTTCACCGACTTT-3’
AMH	5’-AGCAGCTCAACCTCTCTGAGGA-3’	5’-TAGCAGAAAGCCAGAAGGTGC-3’
CYP19A1	5’-CAGCCAGTTGTGGACTTGATCA-3’	5’-TTGTCCCCTTTTTCACAGGATAG-3’
DMRT1	5’-AGCCCAACTCACTCAACAAG-3’	5’-GATGGAAGGAACATCCTGAA-3’
FOXL2	5’-CATCAGCAAGTTCCCCTTC-3’	5’-GGGCACCTTGATGAAACAC-3’
***In situ* probe primer**		
amh	5’- GTGTTTCACCAGGATGACGCCGGTGCT -3’	5’- GGCTCCTCCGACTGCACCAGGCGCTCC -3’

Table S2:

Transient Receptor Potential Vanilloid Receptor subtype 4 homologs in various vertebrates.

Common name	Scientific name	Accession number
American alligator	*Alligator mississippiensis*	LC12707
Chinese alligator	*Alligator sinensis*	XM_006015214.1
Chicken	*Gallus gallus*	NM_204692.1
Turkey	*Meleagris gallopavo*	XM_003210979.1
Pigeon	*Columba livia*	XM_005498200.1
Painted turtle	*Chrysemys picta*	XM_005298531.1
Chinese softshell turtle	*Pelodiscus sinensis*	XM_006130238.1
Green sea turtle	*Chelonia mydas*	XM_007058340.1
Japanese striped snake	*Elaphe quadrivirgata*	AB_666090.1
Japanese grass lizard	*Takydromus tachydromoides*	AB_666089.1
Human	*Homo sapiens*	NM_021625.4
Mouse	*Mus musculus*	NM_022017.3
Rat	*Rattus norvegicus*	NM_023970.1
Japanese treefrog	*Hyla japonica*	AB809362.1
Western clawed frog	*Xenopus tropicalis*	ENSXETT00000040300 (TRPV4a)
Zebrafish	*Danio rerio*	NM_001042730.1
